# BASIC study: is intravaginal boric acid non-inferior to metronidazole in symptomatic bacterial vaginosis? Study protocol for a randomized controlled trial

**DOI:** 10.1186/s13063-015-0852-5

**Published:** 2015-07-26

**Authors:** Melinda Zeron Mullins, Konia M. Trouton

**Affiliations:** Department of Family Practice, University of British Columbia, 3rd Floor David Strangway Building, 5950 University Boulevard, Vancouver, British Columbia BC V6T 1Z3 Canada

**Keywords:** bacterial vaginosis, boric acid, double blinded, intravaginal, metronidazole, multicenter, non-inferiority, placebo, randomized controlled trial

## Abstract

**Background:**

Bacterial vaginosis is associated with increased transmission of sexually transmitted infections, preterm labor, post-surgical infections, and endometritis. Current treatment for symptomatic bacterial vaginosis includes antibiotics, such as metronidazole, which are 70–80 % effective at one month after treatment and result in high recurrence rates and secondary candida infections. Intravaginal boric acid has been used for over a hundred years to treat vaginal infections, such as bacterial vaginosis. Boric acid is inexpensive, accessible, and has shown to be an effective treatment for other infections, such as vaginal candidiasis. To date, there has been no clinical trial evaluation of boric acid effectiveness to treat bacterial vaginosis.

**Methods/Design:**

The BASIC (Boric Acid, Alternate Solution for Intravaginal Colonization) trial is a randomized, double-blinded, multicenter study. The study will enroll a minimum of 240 women of 16–50 years of age who are symptomatic with bacterial vaginosis. Eligible participants will have Amsel and Nugent scores confirming bacterial vaginosis. Women who are pregnant or menopausal or have other active co-infections will be excluded. Consenting participants who meet exclusion and inclusion criteria will be randomly assigned to one of three treatment groups: boric acid, metronidazole, or an inert placebo. Self-administration of treatment intravaginally for 10 days will be followed by clinical assessment at 7 and 30 days (days 17 and 40, respectively) after the end of the treatment phase. Primary outcome is a non-inferiority, per-protocol comparison of the effectiveness of boric acid with that of metronidazole at day 17, as measured by the Nugent score in 16–50 year olds. Secondary outcomes include: non-inferiority, intention-to-treat comparison of effectiveness of boric acid with that of metronidazole at day 17, analysis for both per-protocol and intention-to-treat at day 40, and safety considerations, including adverse effects requiring patient discontinuation of treatment.

**Discussion:**

This study will be the first to determine whether intravaginal boric acid is non-inferior to metronidazole in the treatment of bacterial vaginosis in symptomatic women.

**Trial registration:**

ClinicalTrials.gov NCT00799214, registered online Nov 10, 2008.

## Background

Bacterial vaginosis (BV) is thought to be more common than either vulvovaginal candidiasis or trichomoniasis, and to represent 40–50 % of all cases of vaginitis [1]. Bacterial vaginosis is associated with postpartum endometritis, increased risk of post-surgical infections, pelvic inflammatory disease, increased risk of spontaneous abortions, preterm rupture of membranes and delivery, and increased risk of sexually transmitted infection acquisition, including: human immunodeficiency virus, herpes simplex virus, *Neisseria gonorrhoeae*, *Chlamydia trachomatis*, and *Trichomonas vaginalis* [1–6]. Bacterial vaginosis is caused by a shift from the normal peroxidase-producing lactobacillus-dominant vaginal flora to a several-log increase in total bacterial colony count, which may include a mix of anaerobes, genital mycoplasmas, and *Gardnerella vaginalis* [1, 7]. Bacterial vaginosis is classically characterized by abnormal odor and vaginal discharge in symptomatic women, while 50 % of women with BV are asymptomatic [7, 8]. It can also be painful, and is associated with dysuria or dyspareunia in symptomatic women [8]. Bacterial vaginosis is diagnosed in the presence of three of the four Amsel criteria: elevated pH (>4.5); homogenous milky or creamy discharge; amine odor; and clue cells [4, 7, 9]. The gold standard for BV diagnosis is by Gram staining (Nugent score ≥7), demonstrating a decrease or absence of lactobacilli and the presence of other microbes that cause BV [4, 7, 9, 10].

Untreated women show a 30 % spontaneous resolution rate for BV [11, 12]. Current recommended antimicrobial treatments for BV in non-pregnant, symptomatic women include oral and intravaginal metronidazole or clindamycin [1, 8, 13]. Oral and intravaginal metronidazole are considered equivalent and effective treatments for BV [9, 11, 14–16].

Standard treatment with metronidazole, according to these trials, has imperfect cure rates (approximately 70–80 %) measured 1 month after treatment [9, 11, 12, 15–18] and high BV recurrence risks: 33 % at 3 months [19] and approximately 49–66 % at 1 year [18]. Metronidazole also carries a significant rate of side effects (10–20 % of women), especially with the oral metronidazole preparation [20], including secondary vaginal infection with candida [12, 14, 16]. At present, oral or vaginal metronidazole is the only recommended treatment option for recurrent BV, as is vaginal metronidazole for long-term suppression [9, 21].

Boric acid (H_3_BO_3_) has been used for over a hundred years for the treatment of vaginal infections and is commonly used by physicians and patients as an inexpensive, easy to use, accessible treatment of candidiasis and BV [22]. In addition to its proven effectiveness in the treatment of candidal infections, [22–31] including those that did not resolve with usual antifungal treatment [24–28], H_3_BO_3_ use seemed to be associated with a reduced number of co-infections with BV [25]. A retrospective study suggests clinical improvement in seven of nine patients following treatment with 600 mg intravaginal H_3_BO_3_ for 14 nights in women with a mixed infection of *T. glabrata* vaginitis and BV [25]. In an uncontrolled, non-randomized, retrospective chart review, when H_3_BO_3_ was added to nitroimidazole there was promising long-term (>88 % cure rate at 12 weeks after the study and 50 % at 36 weeks of follow-up) suppression of recurrent BV [32]. Based on the aforementioned study, the physician evidence reference resource ‘UpToDate*®*’ [21] recommends that intravaginal H_3_BO_3_ (600 mg for 21 days) be added to 7 days of metronidazole or tinidazole treatment to induce a remission prior to long-term suppression therapy. No studies to date have determined H_3_BO_3_ effectiveness in the treatment of acute, chronic, or recurrent BV.

The objective of this study was to determine whether intravaginal H_3_BO_3_ is comparable to standard treatment, metronidazole, for the cure of BV in symptomatic women.

### Research question

Among women 16–50 years old symptomatic with BV, is intravaginal treatment with H_3_BO_3_ non-inferior to metronidazole to achieve a Nugent score <7 (cure) by day 17?

### Null hypothesis

*H*_0_: proportion of women cured using H_3_BO_3_ ≤ metronidazole proportion of women cured using metronidazole − 10 %.

## Methods/Design

### Study design

Institutional review board approval was granted for this study through the University of British Columbia Clinical Research Ethics Board (H07-02330). Health Canada Clinical Trial Application Notice of Authorization was obtained on 10 May 2013 (company code 31784, file number 187476). This trial is registered with ClinicalTrials.gov (NCT00799214). This study conforms to CONSORT 2010 guidelines for randomized control trials.

Based on preliminary data, the success rate for the boric acid treatment group is predicted to be approximately 77–88 % [25, 32] in treating BV. Metronidazole would be expected to be 70–80 % [11, 12, 15–18] effective in treating BV, as compared with a 30 % success rate of a placebo group [11, 12]. The Nugent score is found to be >95 % sensitive [33] for detecting BV. We chose a non-inferiority limit of 10 %, as this will allow for a clinically relevant difference between the metronidazole treatment group and the boric acid group. Also, this is approximately 25 % of the expected difference between results obtained for the metronidazole standard treatment group and the placebo group. Accounting for a non-inferiority limit (delta) of 10 %, testing for a significance level of 0.05 and power of 80 %, we require approximately 63 subjects per group. To allow for a dropout rate of 25 %, we set a target rate of 80 subjects per treatment arm. If there is a true difference in favor of the experimental treatment of 8 %, then 126 patients (total for two groups of 63) are needed, to be 80 % sure of the upper limit of a one-sided 95 % confidence interval, using a binary outcome, non-inferiority trial power calculator [34].

We felt it important to include a placebo treatment arm in this study for different reasons: (1) this is the first randomized controlled trial to use intravaginal H_3_BO_3_; thus, comparing it to placebo for safety and effectiveness would be of clinical interest (that is, perhaps it is not comparable to metronidazole in effectiveness but it is still significantly better than placebo), (2) having a placebo group in a non-inferiority trial acts as a sensitivity assay and internal validator for this type of trial [35], (3) comparing metronidazole effectiveness and safety with results obtained for a placebo group in 16–19-year-olds has not previously been done and would also be of clinical interest.

### Study setting

Recruitment began February 2014 for a minimum of 240 volunteers through participating family practice clinics in British Columbia, Canada. Volunteer participating clinics represent a spectrum of different types of practice across British Columbia: rural, remote, urban, First Nations and non-First Nations communities, single and multi-clinician practices, general family practice offices, women’s health clinics, and youth clinics. Clinics were recruited to the study through province-wide medical journal advertisements, university family practice departmental email advertisements, personal emails, faxes, phone calls and in person. Eligible clinics agreed to recruit five or more participants meeting inclusion and exclusion criteria and to use the study treatments and prepared packages provided.

### Participants and eligibility

Women who present to their clinician at participating family practice clinics with symptoms of BV are offered an opportunity to participate in the study. Participants are given the informed consent form explaining the three possible treatment arms, and that participation will include three visits (the initial visit, day 0; day 17–19 [7–9 days after the treatment end to measure the primary endpoint]; and day 40–42 [30–32 days after the treatment end as a secondary endpoint]) involving three examinations, including a pregnancy test. There are no visits to the clinic while the participants are administering the 10-day treatment unless there are any concerns. Women are included in the study if they are symptomatic for BV, meet three of four Amsel criteria on the initial day of the study and subsequently have a swab graded with a Nugent score ≥7. Detailed eligibility criteria are listed in Table 1. In general, participants cannot be pregnant, menopausal, have another vaginal infection, or have an intrauterine device. Written consent is obtained prior to treatment intervention. Women who choose to participate receive their medication for free. There is a draw for recruited women to win a small gift certificate.

**Table 1:** Eligibility criteria

Inclusion criteria	Exclusion criteria
Must be 16–50 years old and pre-menopausal. Capable of giving written informed consent.	Younger than 16 or post-menopausal.
Fluent spoken and written English.	Menstruating at diagnosis.
Agrees to examination on enrollment day, day 17–19 and day 40–42.	Symptoms so severe as to make allocation to placebo unacceptable to the woman.
Has a negative pregnancy test during the study. Agrees to follow study protocol and is reliable for follow-up.	Currently pregnant or at high risk for pregnancy.
Has documented bacterial vaginosis infection by positive vaginal swab (minimum Nugent score of ≥7) and meets three of four Amsel criteria.	Current active sexually transmitted infection (chlamydia, gonorrhea, trichomonas).
Agrees to refrain from vaginal intercourse for the 10 days of treatment (or to use non-lubricated non-latex condoms if unavoidable). Agrees not to douche or use any intravaginal products during treatment (including tampons, medications, devices).	Current yeast infection as determined by history, physical examination, and swab analysis.
Agrees to abstain from alcohol during the 10 days of treatment (from 24 hours before through 72 hours after taking study medication).	History of pelvic inflammatory disease. Allergy to metronidazole.
Agrees to no new medications or antibiotics during treatment.	Currently breast feeding.
Has no currently active sexually transmitted infection as determined by history, physical examination, and swab analysis negative for chlamydia, gonorrhea, candidiasis, or trichomonas.	Any open wound, excoriation, or vaginal irritation, including Bartholin’s cyst, abscess, and herpes simplex viral lesion, as determined by physical exam.
	Presence of another vulvar, vaginal or medical condition, including cervical neoplasia or treatment, or medical device, including intrauterine device, that might confound treatment response.
	Using lithium, anticoagulants or disulfiram drugs.
	Any antifungal or antibiotic use 14 days prior to enrolment.
	Papanicolaou smear taken within one week of enrolment.
	Meeting three of four Amsel criteria but having a Nugent score <7.

### Randomization

Randomization in blocks of 80 was conducted by the study pharmacist (who was blinded to enrollment and outcome measurement) using the ‘first generator’ on the website: www.randomization.com in September 2013. These numbers then become the new identification numbers on the treatment packs. All treatment packs are then distributed to participating clinics by the study pharmacist who sends out five sequentially numbered treatment packs to each participating site as sites are recruited to the study. The study pharmacist is the only person with access to the master code to the randomized treatments. The participants, recruiting clinicians and investigators will be blinded to the controlled and randomized 10-day treatment pack that is given to the study participant. Blinded recruiting clinicians give out the physically indistinguishable treatment package in allocated randomization order to the sequential participants on enrollment day (day 0). Subsequently the treatment pack number becomes the participants’ identification number.

### Intervention and control groups

On day 0, the enrolled participants will be blindly and randomly assigned to one of three 10-day treatments (minimum of 80 women per treatment arm): (1) placebo (emollient cream); (2) H_3_BO_3_ (600 mg H_3_BO_3_ compounded in emollient cream); (3) metronidazole (10 %, for a total of 37.5 mg metronidazole) intravaginal cream (Sanofi-Aventis Canada Inc. Product DIN 01926861) packaged by the study pharmacist. There is no detectable difference between the treatments in either appearance or scent.

### Study procedure and data collection

On day 0, a pregnancy test will be conducted and history will be collected by the clinician. During a pelvic examination, the clinician will ensure intact mucous membranes and anatomy, observe whether there is homogenous milky or creamy discharge (one of the four Amsel criteria), take swabs for chlamydia and gonorrhea, BV (for the Nugent score), candidiasis, and trichomonas, and perform a Whiff test using a provided standardized 10 % potassium hydroxide solution (one of the four Amsel criteria). Vaginal discharge will also be tested for pH (one of the four Amsel criteria). Where facilities permit; clue cells will be examined, as the fourth Amsel criterion. On this day, the recruiting clinician will confirm participation, eligibility, and exclusion criteria using provided protocol forms and will collect the signed consent form and the demographic and medical history questionnaires provided.

The blinded treatment pack (containing either H_3_BO_3_ compounded in emollient cream, metronidazole cream, or emollient cream) is allocated by the recruiting clinician in the randomization order to sequential participants on day 0. The participant package also contains: (1) vaginal applicator; (2) pads; (3) non-lubricated non-latex condoms; (4) a diary to record daily use of the treatment (5) information sheet outlining follow-up instructions and emergency contact numbers for the study members. Participants will self-administer the blinded treatment cream intravaginally each evening immediately prior to sleep for 10 days. The participants will be instructed to use a provided pad during day and night. Each participant will record daily compliance, side effects, and symptoms in the journal provided.

On both follow-up visits: days 17–19 (one week after the treatment end) and 40–42 (one month after the treatment end), the participant will return to the clinic that enrolled her for reassessment, a pregnancy test, and a follow-up examination, including a pelvic examination and repeat swab analysis. It will be noted whether her BV symptoms are still present, and if she had any side effects or problems during treatment. The participant will also return her daily treatment diary on the day 17 visit.

### Safety assessments

Emergency telephone contact with the investigators will be available for any questions or problems related to the treatment. On day 5 of the treatment, the participant will receive a call, text, or email from one of the investigators to determine and record compliance, side effects, BV symptoms, and satisfaction with treatment. The study pharmacist will be informed by the recruiting clinician of the participant’s full name, date of birth, and personal health number, and the study number assigned, so that the study pharmacist can enter the medication name into the British Columbia PharmaNet Database (the provincial pharmacy database). This safety measure ensures that subsequent treating physicians throughout the province will have access to details of the treatment received, in case of a safety concern and a need to unblind the participant’s medication should the participant present with an emergency. Non-emergency access to the provincial pharmacy database is not allowed, to protect the participant’s identification of the blinded treatment.

If at any time the participant reports intolerable side effects (vaginal discomfort, intolerable vaginal discharge, urticaria, or secondary yeast infection) during treatment, or exacerbation of symptoms at any point, she may voluntarily discontinue the treatment and leave the study without prejudice, and her doctor or another physician may provide treatment as per usual standard of care.

As neither metronidazole nor H_3_BO_3_ treatments provided to patients are in amounts considered toxic when taken intravaginally or if accidentally ingested, should there be any occurrence of serious adverse effects (neurological, cardiac, respiratory, gastrointestinal, or anaphylactic reactions) during any of the treatments, the study will be put on hold until an investigation can be held. In the case of serious adverse effects found to be caused by the treatment, the study will be stopped, and the study unblinded and decoded by the study pharmacist. The investigators, participants, and clinicians involved in the study would be informed for the reason for stopping the study.

### Outcomes and assessment

On days 17–19 and days 40–42, if the vaginal swab is positive for BV found by both meeting three of four Amsel criteria and having a Nugent score ≥7 and the participant is symptomatic for BV, the physician will prescribes a standard medication of his choice. This is considered a treatment failure addressing our primary and secondary outcomes: effectiveness at 7 days and 30 days after treatment end.

If the participant discontinued the treatment during the 10 days because of side effects or complained of intolerable side effects (major adverse effects) from the treatment, this is considered a treatment failure referring to our secondary outcome: safety.

### Statistical considerations

The primary outcome measure is a non-inferiority per-protocol comparison of effectiveness of H_3_BO_3_ with metronidazole at day 17, as measured by Nugent score, in 16–50-year-olds with a *z*-based confidence interval for the difference of two proportions. This was chosen as the primary outcome measure because the immediate effect of H_3_BO_3_ after a 10-day treatment compared with metronidazole was our most important question about BV treatment. A change from a baseline Nugent score ≥7 to a Nugent score <7 is considered a cure of BV. Treatment failure will be reported as a positive Nugent score ≥7 at day 17.

Secondary outcome measures include a non-inferiority intention-to-treat comparison of effectiveness of H_3_BO_3_ with metronidazole at day 17, as measured by Nugent score in 16–50-year-olds, with a *z*-based confidence interval for the difference of two proportions. We will use this comparison to confirm the robustness of our per-protocol comparison but the per-protocol analysis will be the primary determinant of the non-inferiority test. In addition, we will conduct a non-inferiority comparison of effectiveness of H_3_BO_3_ and metronidazole at day 40, as measured by Nugent score in 16–50-year-olds analyzed on both per-protocol and intention-to treat principles, with a *z*-based confidence interval for the difference of two proportions, and safety consideration including intolerable adverse effects requiring patient discontinuation of the 10-day treatment. To control for multiple testing, we will use a Bonferroni-type correction. To account for both intention-to-treat and per-protocol analyses, and two timepoint analyses, the threshold for statistical significance will be set at 0.01 (approximately 0.05 divided by 4).

The following are tertiary outcome measures and no formal sample size calculations were made. Minor adverse effects (side effects not causing the patient to discontinue treatment) will be reported and compared between the treatment arms but will not be considered as treatment failure. We will examine the adolescent population, the 16–19 year old subgroup, for non-inferiority for effectiveness of H_3_BO_3_, as compared with metronidazole, at the end of the study. Finally, we will test for superiority of the effectiveness and safety of H_3_BO_3_ compared with placebo in both the 16–50-year-olds and in the 16–19 year old subgroup. In addition, our study is the first to be able to examine 16–19-year-olds for the effectiveness and safety of metronidazole compared with placebo.

95 % confidence intervals will be established. Missing data (participants who fail to complete follow-up for any reason) are accounted for by an anticipated 25 % lost-to-follow-up rate. Data will be blindly collected by the outcome assessors from the participating clinics and entered into an electronic database. The study pharmacist will provide the randomization code for the data to a third-party statistician and the data will be analyzed. Self-reported participant satisfaction criteria included at the end of the daily treatment diary will use a five-point rating scale to record: effectiveness in curing BV symptoms, side effects, ease of use, likelihood they would use it again for BV treatment, likelihood they would recommend it to someone else for BV treatment.

## Discussion

The BASIC (Boric Acid, Alternate Solution for Intravaginal Colonization) study will be the first prospective randomized control trial to determine the effectiveness of intravaginal H_3_BO_3_ compared with metronidazole for the treatment of BV in symptomatic women. If intravaginal H_3_BO_3_ is shown to be non-inferior to metronidazole in the treatment of BV, this may provide women with less costly options for treating this common vaginal infection.

## Trial status

The trial is currently open to recruitment.

## Abbreviations

BASIC: Boric Acid, Alternate Solution for Intravaginal Colonization
BV: bacterial vaginosis
